# How Different Carryover Pitch Extractive Components are Affecting Kraft Paper Strength

**DOI:** 10.1021/acsomega.1c02579

**Published:** 2021-10-26

**Authors:** Jussi Lahti, Roman Poschner, Werner Schlemmer, Andrea Hochegger, Erich Leitner, Stefan Spirk, Ulrich Hirn

**Affiliations:** † Institute of Bioproducts and Paper Technology, Graz University of Technology, Inffeldgasse 23, Graz 8010, Austria; ‡ Christian Doppler Laboratory for Fiber Swelling and Paper Performance, Boltzmanngasse 20/1/3, Vienna 1090, Austria; § Institute of Analytical Chemistry and Food Chemistry, Graz University of Technology, Stremayrgasse 9/II, Graz 8010, Austria

## Abstract

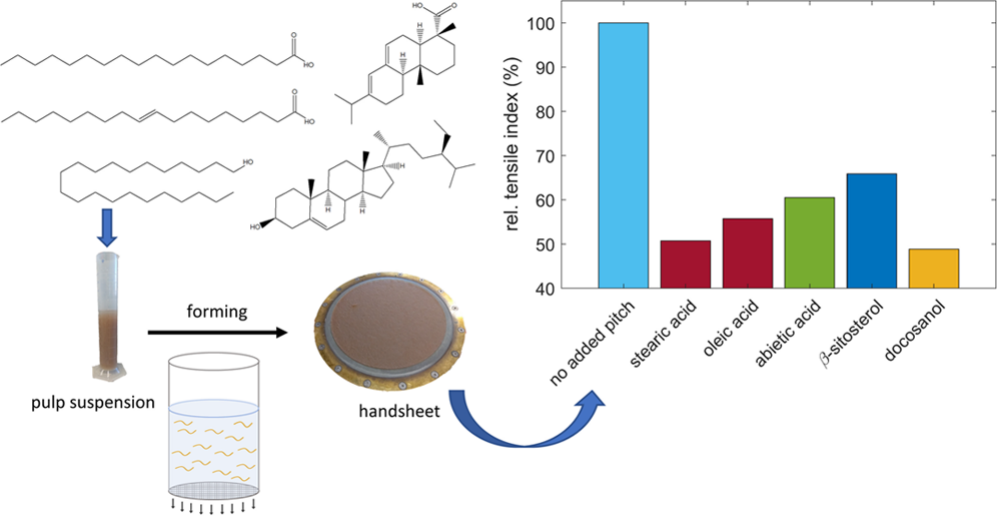

We present how harmful different wood extractives carried over to paper mill with unbleached softwood Kraft pulp are for the strength of packaging papers and boards. The investigations were done by simulating industrial papermaking conditions in laboratory-scale trials for handsheet production. It was found that fatty acids are the most relevant compounds in the carryover pitch extractives (CPEs), as they readily interfere in fiber–fiber bonding strength, control the properties of CPE micelles, and are furthermore the most abundant compounds. Addition of cationic starch improved strength and evened out the strength differences of handsheets with different CPE compounds. Oleic acid (unsaturated fatty acid) was an exception, as it was above average harmful for paper strength without cationic starch and also heavily impaired the functioning of cationic starch. As a whole, these findings demonstrate that fatty acids, especially unsaturated ones, are the most relevant CPE compounds contributing to the reduced efficiency of cationic starch and decreased strength of unbleached softwood Kraft paper. This makes the cleaning of process waters by precipitating CPEs on the pulp fibers harmful for paper strength.

## 1 Introduction

Wood extractives (also called pitch) is a common term for different types of compounds consisting of triglycerides, fatty acids, steryl esters, and sterols present in soft- and hardwoods. Besides, resin acids are also present in softwoods.^1,2^ In paper production, they are relevant unwanted substances, as they cause various operational efficiency and paper quality issues during paper manufacturing.^3−7^ In the course of Kraft pulping for instance, while liberating the pulp fibers through delignification, extractives are separated under hot alkaline conditions where a part of the extractives and carbohydrates degrade and dissolve. Subsequent washing steps remove the extractives from the pulp to a large extent. However, some extractives are always carried over to the paper mill, as washing with hot alkaline water is insufficient for their complete removal. These extractives are therefore called carryover pitch extractives (CPEs).^4,7,8^

**Figure 1:**
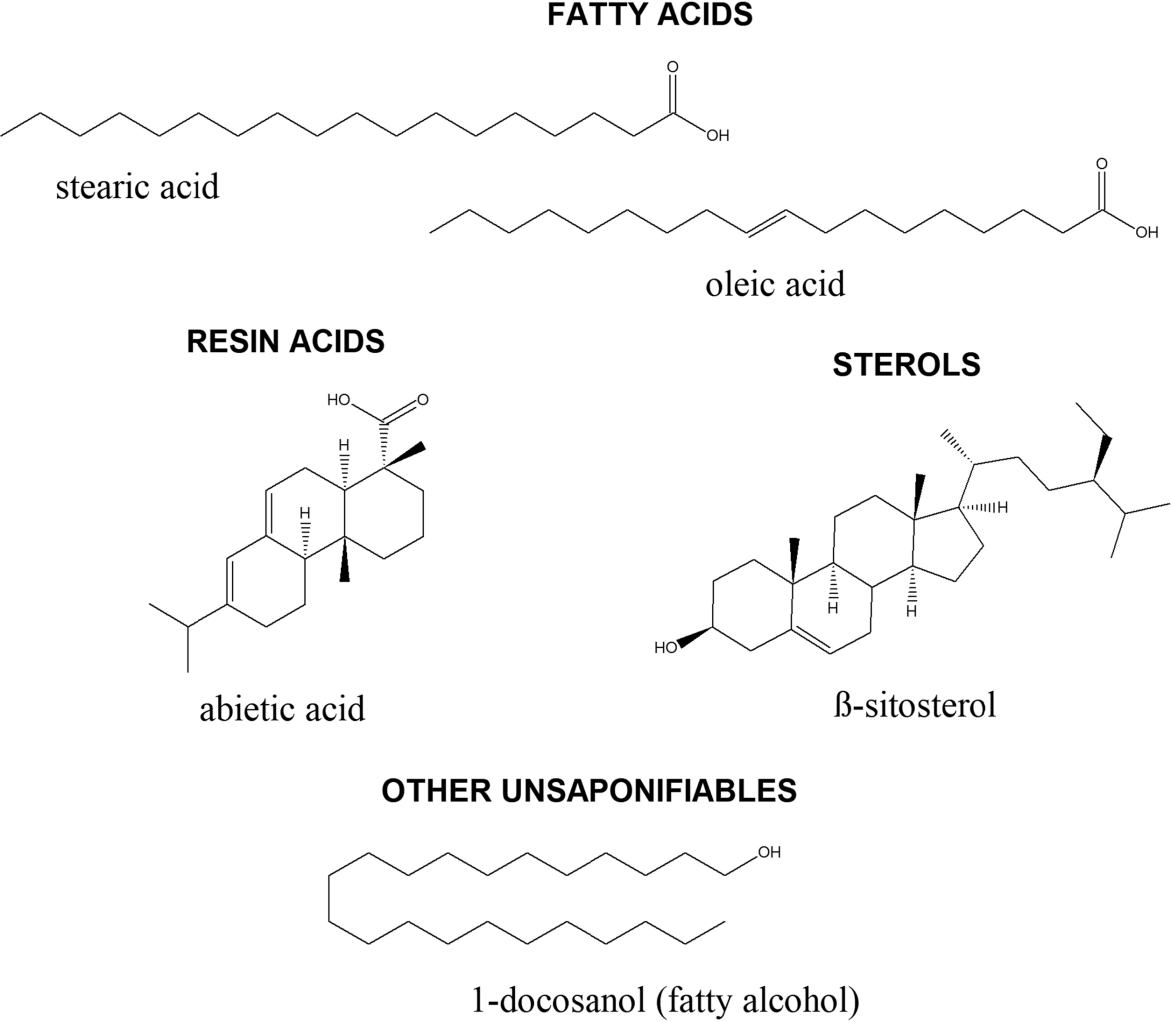
Examples of pitch compounds from the major classes of carryover pitch extractives.

The composition of CPEs differs from that of wood extractives, as alkaline hydrolysis during Kraft cooking induces chemical changes to some, while others are removed by the alkaline treatment. Consequently, CPEs include fatty and resin acids, sterols, and other unsaponifiables, such as triterpenyl alcohols, diterpene aldehydes and alcohols, fatty alcohols, and terpenoid hydrocarbons.^1,2,8^ Examples of these compound classes are shown in Figure 1.

Due to their (at least partial) hydrophobicity, CPEs have a tendency to form deposits in the papermaking process loops, requiring regular cleaning and maintenance. The saponifiable CPE compounds (fatty/resin acids) in turn potentially cause foaming of the circulating process waters and thus reduce the operational efficiency of a paper machine. Furthermore, the presence of CPEs reduces paper strength by complex formation of anionic fatty/resin acids with cationic dry strength agents (e.g., cationic starch), which leads to the requirement of costly additional dosage of the cationic dry strength agent. To prevent these problems from occurring, the circulating process waters need to be cleaned by adsorbents and cationic retention aids such as aluminum sulfate (alum), which precipitates the CPEs onto the pulp fibers. However, the CPE precipitates on the fiber surface impart fiber–fiber bonding and thus are detrimental for paper strength as well.^7,9−16^

As softwood Kraft pulp is often used to reinforce packaging papers/boards, the identification and removal of strength-reducing CPEs is of utmost importance to address the need for light weight, high-strength packaging materials.^17^ While there is a multitude of studies on the detrimental effect of pitch on paper strength,^12,16,18−23^ there are no studies on the effect of CPEs or their individual compounds on the strength of unbleached softwood Kraft paper. Sundberg et al.^12^ observed that the strength of bleached softwood Kraft paper decreased strongly when wood extractives were precipitated onto the fibers by cationic retention aids. Brandal and Lindheim^18^ and Kokkonen et al.^20^ in turn concluded that individual wood extractive compounds with long linear hydrocarbon chains are most harmful for the strength of mechanical papers. We extend these previous studies by examining the abundance of different softwood, i.e., a mixture of spruce and pine, CPE compounds, and how the alum-assisted precipitation of these compounds onto the unbleached softwood (mixture of spruce and pine) Kraft fibers influences the paper strength. Moreover, it is investigated how this alum-assisted precipitation of different CPE compounds influences the functioning of cationic starch as a dry strength agent.

To facilitate these tasks, the abundance of major softwood CPE classes (see Figure 1) was first analyzed by two-dimensional gas chromatography-mass spectrometry and then model compounds from each class were used in laboratory paper production. The model compounds were dispersed and added to white water for laboratory paper production, with industrial process conditions being emulated as accurately as possible. The effect of alum-assisted retention of the different CPE model compounds on paper properties is studied and compared to the effect of alum-induced retention of industrial carryover pitch collected from the filtrate of the pulp entering the paper mill. Finally, the effect of alum-assisted retention of CPE model compounds as well as industrial carryover pitch on cationic starch with respect to paper strength improvement is evaluated.

## 2 Experimental Section

### 2.1 Analysis of Carryover Pitch Extractives

Industrial softwood, i.e., a mixture of spruce and pine, CPEs were provided by a European Kraft pulp mill. They were acetone extracted from the produced unbleached softwood (mixture of spruce and pine) Kraft pulp after the last pulp washing step according to ISO 14453 (ISO standards are described in the Supporting Information). The direct measurement of the CPE compounds adsorbed to the fibers by ATR-IR spectroscopy is difficult for small quantities.^24^ A more simple method to analyze pitch components is 2D thin-layer chromatography (TLC).^25^ We carried out two-dimensional gas chromatography-mass spectrometry (2D-GC×GC-MS), which is more elaborate. Therefore, 10 mg of CPEs were dissolved in 1 mL of acetone, and from this solution, a sample volume of 100 μL was taken for analysis. After drying the sample in a N_2_ stream, it was silylated by adding 50 μL of BSTFA+TMCS and 50 μL of pyridine (puriss. p.a., ACS; ≥99.8%; Fluka, Switzerland) and stirred for 30 min at room temperature. Prior to the analysis, 900 μL of ethyl acetate (Picograde for Residue Analysis; LGC Promochem, Germany) was added. The comprehensive GC×GC-MS was composed of the following units: an OPTIC-4 Multimode GC inlet, an AOC-5000 Plus autosampler, and a Shimadzu gas chromatograph-mass spectrometer GC-2010 Plus equipped in the first dimension with a Rxi-1HT column (30 m × 0.25 mm; 0.25 μm) and a Rxi-17SilMS column (2 m × 0.15 mm; 0.15 μm) in the second dimension, both were placed in the same oven. The columns were connected directly using an Agilent Ultimate Union Kit. The injection was done in the splitless mode; the split was opened after 2 min with a split ratio of 5. The injector port temperature was 270 °C. Helium was used as a carrier gas in the linear velocity flow control mode. The pressure was set to 90 kPa and the purge flow was 6.0 mL min^–1^. The initial oven temperature of 50 °C (30 min) was increased to 300 °C (0 min) with a ramp of 5 °C min^–1^ and then to 320 °C (3 min) with a ramp of 20 °C min^–1^. The modulator used was a cryogenic modulator (Zoex ZX1 two-stage loop thermal modulator; Zoex Corporation, Houston, Texas). The modulation frequency was 5 s with a hot jet pulse of 500 ms. The temperature of the hot jet was programmed at 340 °C (40 min), followed by 360 °C for 5 min, and finally set to 280 °C till the end of the run. Detection was done using the mass spectrometer GCMS-QP2010 Ultra. Ions were generated with electron ionization (70 eV). The ion source temperature was set at 200 °C, the interface temperature was 310 °C, and the detector voltage was 1.1 kV. The mass spectrometer was scanned with a scan speed of 20 000 u s^–1^, resulting in 33 full scan spectra recorded from *m*/*z* 55 to 500. The solvent delay was 4 min, i.e., the data acquisition was initiated after 4 min runtime and ended after 52 min runtime. The software used for data evaluation was version 2.7. of “GC Image” from Zoex Corporation.

### 2.2 Laboratory Papermaking Trials

Model compounds (purity ≥ 80%; Merck, Darmstadt, Germany) from each CPE class shown in Figure 1 were selected for laboratory papermaking, investigating the effect of each individual softwood pitch compound on the performance of unbleached softwood Kraft paper. In earlier investigations, it was found that saturated and unsaturated fatty acids had a different effect on the strength of thermomechanical pulp (TMP) handsheets.^20^ Thus both unsaturated (oleic acid) and saturated (stearic acid) fatty acids were tested in this trial. Fatty alcohol (docosanol) was in turn selected from the class of other unsaponifiables, as they have been found to be especially harmful for the strength of groundwood handsheets.^18^ Industrial carryover pitch from softwood (mixture of spruce and pine) was additionally tested as a reference to enable better evaluation of the harmfulness of individual pitch compounds. It was collected together with the other dissolved carryover compounds, i.e., lignin, carbohydrates, and inorganics,^8^ by drying the filtrate of the unbleached softwood Kraft pulp (provided by a European softwood Kraft pulp mill) entering the paper mill after the last pulp washing step. This filtrate had a chemical oxygen demand of 2500 mg L^–1^, a CPE content of 50 mg L^–1^, and an inorganic content of 356 mg L^–1^.

**Figure 2:**
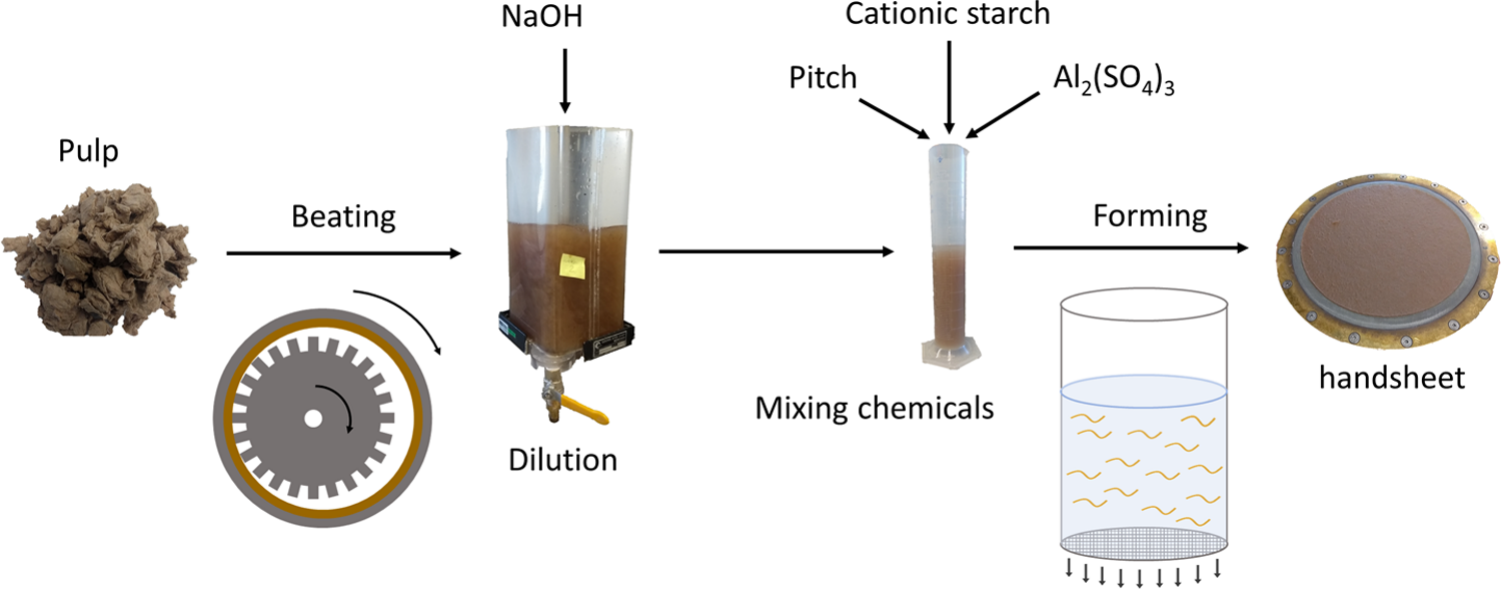
Schematic for preparing handsheets to investigate the effect of carryover pitch extractives on Kraft paper performance.

Figure 2 shows the schematic for investigating the influence of CPEs on Kraft paper performance. Never-dried unbleached softwood (mixture of spruce and pine) Kraft pulp with a kappa number of 45 was provided by the European Kraft pulp mill. The pulp was first beaten according to ISO-5264-2 (ISO standards and number of replicates are described in the Supporting Information) in a PFI mill for 3000 rev to reach a Schopper Riegler (°SR) degree of 15 (ISO 5267-1). Handsheet preparation from this beaten pulp was performed according to ISO 5269-2 (rapid Köthen method). The pulp was first diluted with tap water to a concentration of 3 g L^–1^ (oven-dry basis). The pH of this fiber suspension was then adjusted to 10 with NaOH to mimic the alkaline conditions of the industrial papermaking process after beating. Subsequently, 783 mL of the prepared fiber suspension was poured into a beaker. Pitch model compounds, industrial carryover pitch, and process chemicals were prepared and added to the suspension as described below. Finally, the handsheets were formed and dried according to the rapid Köthen method.

#### 2.2.1 Dispersions of Pitch Model Compounds

The pitch model compounds shown in Figure 1 and industrial carryover pitch were added to the fiber suspension as a dispersion. The industrial carryover pitch was added together with the other collected carryover compounds, i.e., lignin, carbohydrates, and inorganics. The procedure for preparing these dispersions was adapted from the method developed by Sundberg et al.^26^ First, 24 mg of each model compound or industrial carryover pitch (together with the other collected carryover compounds) was dissolved in 10 mL of acetone. This solution was then added dropwise to 600 mL of water with pH 8–9 (deionized water with NaOH) under intensive stirring. The concentration of the pitch was 40 mg L^–1^, and the formed dispersion was intensively stirred under ambient conditions to evaporate acetone. Finally, 5 min ultrasonic mixing was applied to stabilize the dispersion. Complete removal of acetone from the dispersion through dialysis was not necessary as it has been found earlier^23^ not to influence the deposition behavior of pitch on pulp fibers (TMP) over a wide range of added pitch concentrations (0–30 mg/g pulp). An amount of 1 mg of pitch model compound or industrial carryover pitch (together with the other collected carryover compounds) per gram pulp (oven-dry basis) was added in the fiber suspension followed by stirring for 1 min. The amount of pitch present in the pulp used in this study was 0.8 mg/g pulp. In other words, the maximum possible amount of pitch model compound or industrial carryover pitch in the prepared handsheets was 1.8 mg/g pulp. According to Laine et al.,^27^ the pitch content of Kraft pulp was below 5 mg/g pulp. Thus, the amount of pitch added in the current study was within the industrially relevant range.

#### 2.2.2 Cationic Starch Solution

Cationic potato starch (Cationamyl 9853K, Agrana) with a low degree of substitution (0.029–0.035) was used as a dry strength agent. A solution was prepared by adding 2 g of cationic starch to 200 mL of deionized water, i.e., the concentration of starch was 10 g L^–1^. To dissolve the starch, the water–starch mixture was cooked at 90 °C for 20 min under stirring. Deionized water was added during cooking to compensate for the evaporation loss. A cationic starch amount of 15 mg/g pulp (oven-dry basis) was added to the fiber suspension followed by stirring for 1 min.

#### 2.2.3 Alum Solution

Alum is very often used as a fixating agent or retention aid in papermaking. In the current study, alum from Kemira Oyj was used. A solution was prepared by first dissolving 30 g of alum in 500 mL of deionized water under ultrasonic mixing. Further dilution with deionized water to a concentration of 30 g L^–1^ was then performed. This solution was added to the fiber suspension to reach pH 6.8 followed by stirring for 1 min.

### 2.3 Paper Testing

The prepared handsheets were first conditioned for 24 h in a standard climate of 23 °C and 50% relative humidity (ISO 187). The basis weight of the handsheets was then measured according to ISO 536 (ISO standards and number of replicates are described in the Supporting Information). ISO 534 was in turn utilized to determine thickness and density. The tensile properties for the handsheets were measured according to ISO 1924-3. Furthermore the internal bond strength of the handsheets was evaluated as Scott bond energy (SBE), i.e., the delamination resistance of paper (ISO 16260).^28^ Finally, Soxhlet extraction of handsheets was conducted with acetone to gravimetrically measure the pitch content of the pulp (ISO 14453).

## 3 Results and Discussion

### 3.1 Composition of Carryover Pitch Extractives (CPEs)

**Figure 3:**
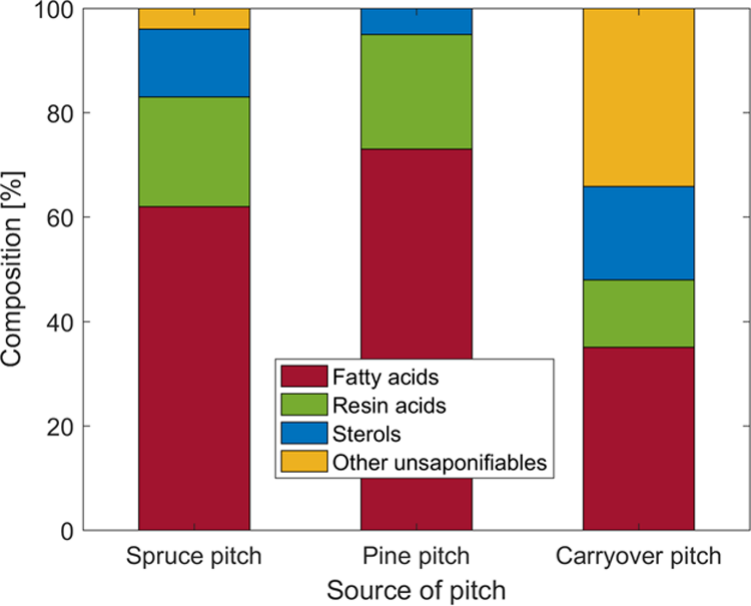
Composition of hydrolyzed pitch from softwood, spruce (left) and pine (middle), according to Vikström et al.^2^ The composition of pitch carried over to the paper mill after Kraft pulping is different (see text), e.g., for the softwood (mixture of spruce and pine) Kraft pulp used in this study (right).

Figure 3 shows the GC-MS measured composition of hydrolyzed softwood pitch of spruce and pine (left and middle, respectively) and the softwood (mixture of spruce and pine) Kraft pulp pitch used in this study (right). The Kraft pulping changes the composition of pitch. Fatty acid and resin acid contents decrease due to Kraft pulping, as their deprotonation favors solubility in water and subsequent removal in pulp washing. As a consequence, the relative amounts of sterols and other unsaponifiable compounds increase. The removal of unsaponifiables is particularly challenging to address, as they dissolve into micelles formed by saponifiables, resulting in stable colloids, impeding their separation.^8,29−34^ In the following, the harmfulness of pitch model compounds from the major CPE classes (see Figure 1) on the strength of unbleached softwood Kraft paper is discussed.

### 3.2 Influence of Individual Pitch Compounds on the Strength of Unbleached Softwood Kraft Paper

**Figure 4:**
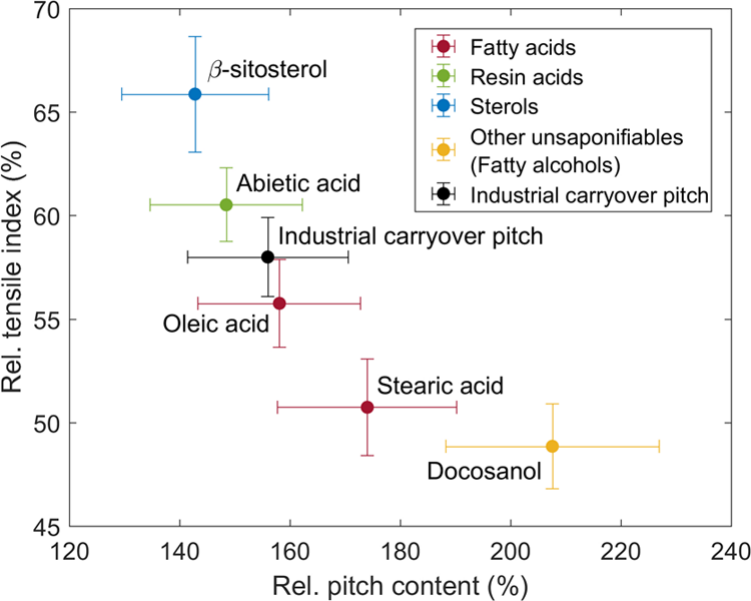
Influence of pitch added to the papermaking suspension on paper strength: pitch content (PC) in handsheet versus tensile index (TI). The values with the addition of pitch are normalized against the values without the addition of pitch, i.e., PC_pitch added_/PC_pitch not added_ and TI_pitch added_/TI_pitch not added_. Error bars are 95% confidence limits.

Figure 4 shows paper tensile strength over pitch amount in the paper for different pitch components and industrial carryover pitch collected from the filtrate of the pulp entering the paper mill. Please note that the values for both pitch amount and paper strength are normalized by the respective values without the addition of pitch before papermaking. The tensile index (TI) and pitch content of paper without the addition of pitch were 83.9 ± 1.6 Nm g^–1^ (95% confidence limits) and 0.79 ± 0.05 mg/g pulp (oven-dry basis), respectively. The retention of all pitch compounds strongly decreased the tensile strength of the prepared handsheets (Figure 4). The TI also decreased by 42% for the industrial carryover pitch with increasing pitch content of 56% (0.44 mg/g pulp). The other compounds, i.e., lignin, carbohydrates, and inorganics, added together with the industrial carryover pitch might have had an influence as well. Koljonen et al.^21^ found that the precipitation of pitch together with lignin and inorganics onto the softwood Kraft pulp fibers impaired paper strength. On the other hand, Sundberg et al.^12^ reported a decrease in the harmful effect of wood pitch on bleached softwood Kraft paper in the presence of carbohydrates. In any case, our result with industrial carryover pitch is in good agreement with Sundberg et al.^12^ In that report, the TI of bleached softwood Kraft pulp handsheets decreased by almost 50% when the wood pitch content in the handsheets was increased with the aid of different cationic retention aids from 0.1 to 2 mg/g pulp. The same study found a much lower pitch retention when preparing paper without retention aids, which for laboratory studies highlights the importance of using a retention/fixation agent (e.g., alum) similar to the case in the industrial papermaking process.

The influence of individual pitch model compounds on paper strength considerably varied in the trials. The linear pitch compounds (oleic acid, stearic acid, and docosanol) adsorbed/precipitated more efficiently on the pulp fibers and led to a lower tensile index in comparison to industrial carryover pitch. Cyclic compounds (β-sitosterol and abietic acid) in turn decreased the TI less than industrial carryover pitch and they precipitated less efficiently on the pulp fibers. The strength results described here are in agreement with those of Brandal and Lindheim^18^ who found out that unsaturated fatty acids (oleic and linoleic acid) and fatty alcohol (*n*-dodecyl alcohol) are more harmful for the tensile strength of groundwood handsheets than, for example, resin acid (abietic acid). Kokkonen et al.^20^ also stated that unsaturated fatty acid (oleic acid) is more harmful for the tensile strength of TMP handsheets than sterol (β-sitosterol) and resin acids. However, they also concluded that the influence of saturated fatty acid (stearic acid) on the tensile strength was negligible, which is contrary to our observations with unbleached softwood Kraft paper.

By combining the results from Figures 3 and 4, it can be concluded that the portion of fatty acids from CPEs is the highest, precipitation of fatty acids on pulp fibers is fairly efficient and they are harmful for the TI of Kraft paper. Furthermore, the surface-active fatty acids together with resin acids tend to form micelles in which the unsaponifiables dissolve. The fatty and resin acids control the properties of these micelles,^35^ and thus their contribution to the negative effect of CPEs is enhanced. Consequently, fatty acids are the most relevant CPEs contributing to the decrease in the TI of unbleached softwood Kraft paper.

The density of paper without pitch was 658 ± 14 kg m^–3^ (95% confidence limits). The alum-assisted retention of pitch compounds did not influence the density of the prepared handsheets (see Figure S1 in the Supporting Information). This means that the fiber surface available for bonding to other fibers is equivalent for the different pitch substances and, more importantly, also essentially the same like in paper prepared without adding pitch. On the other hand, Sundberg et al.^12^ found a slight decrease in density (up to 10%) of the bleached softwood Kraft pulp handsheets, independent of the retained wood pitch content (varied between 0.4 and 10.2 mg/g pulp). Kokkonen et al.^20^ in turn observed that the density of TMP handsheets decreased with increasing retention of oleic acid or resin acids. These findings indicate that the precipitation of pitch on the fibers may also reduce paper strength through the reduction of fiber–fiber bonding area.

**Figure 5:**
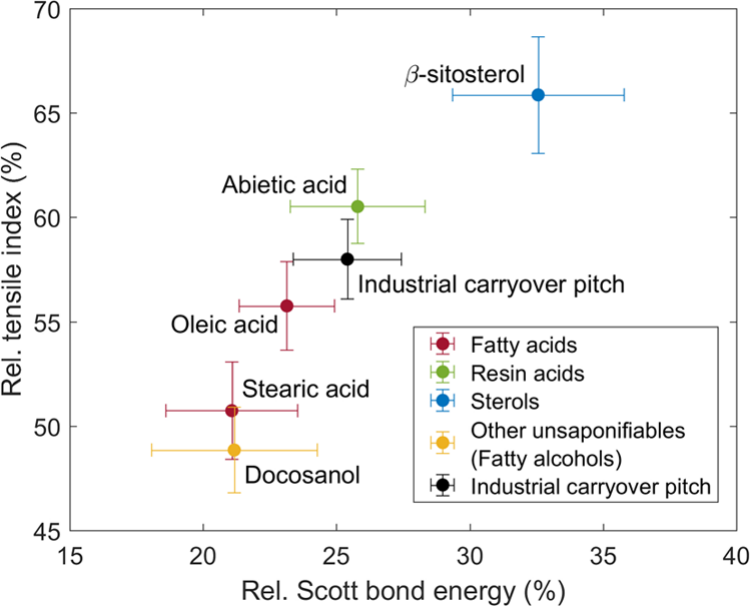
Influence of pitch retention on Kraft paper: Internal bond strength (Scott bond energy, SBE) of paper versus tensile index (TI). The values with the addition of pitch are normalized against the values without the addition of pitch, i.e., SBE_pitch added_/SBE_pitch not added_ and TI_pitch added_/TI_pitch not added_. Error bars are 95% confidence limits.

Figure 5 shows that the internal bonding strength of the paper (SBE) drastically decreased (68 to 78%) together with tensile strength due to the retention of pitch compounds. The SBE of paper without pitch was 442 ± 32 J m^–2^ (95% confidence limits). As in the case of tensile strength, the linear pitch compounds (oleic acid, stearic acid, and docosanol) possessed lower SBE in comparison to the cyclic pitch compounds (β-sitosterol and abietic acid). Both the SBE and TI with industrial carryover pitch were approximately in between these two groups. Together with the fact that the paper densities remained constant, this actually proves that the loss in tensile strength by CPEs is caused by the reduction of the fiber–fiber bonding strength: a decrease of paper internal bonding strength was found without any change in fiber–fiber bonding area (i.e., constant density). As the paper rupture with/without pitch compounds was dominated by the failure of fiber–fiber bonds, the fibers were pulled out from the paper structure during the rupture propagation, i.e., fibers themselves did not break (see Figure S2 in the Supporting Information). Sundberg et al.^12^ also found a clear decrease in SBE (up to 22%) of the bleached softwood Kraft pulp handsheets with increasing wood pitch retention (up to 10.2 mg/g pulp). However, in this case, due to the slightly decreased paper density, the loss in tensile strength was probably partly caused by the reduced fiber–fiber bonding area in addition to the reduced fiber–fiber bonding strength. In any case, it is clear that the precipitated layer of pitch on the fibers prevents fiber–fiber bonding to properly take place.

**Figure 6:**
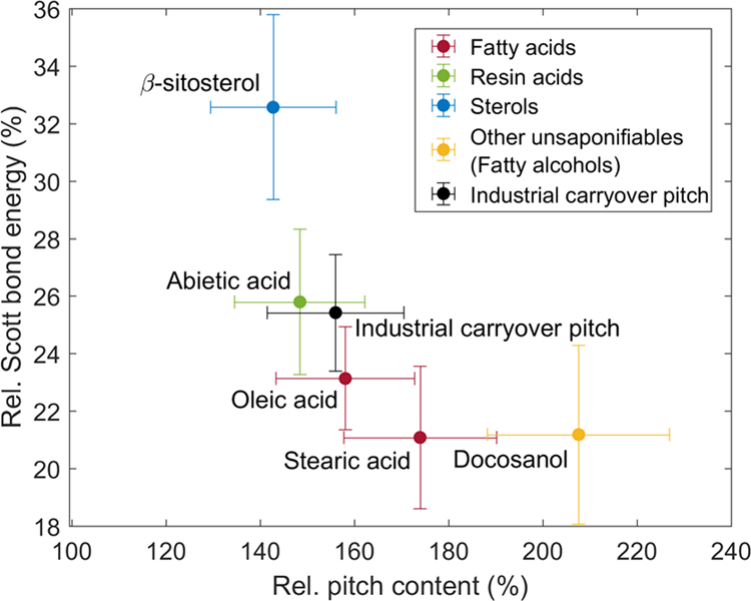
Influence of pitch content (PC) on internal bond strength (Scott bond energy, SBE) of Kraft paper. The values with the addition of pitch are normalized against the values without the addition of pitch, i.e., PC_pitch added_/PC_pitch not added_ and SBE_pitch added_/SBE_pitch not added_. Error bars are 95% confidence limits.

The fiber–fiber bonding strength decreased not only due to increased pitch retention but also due to the pitch compound structure, as illustrated in Figure 6. Linear pitch compounds stearic acid and docosanol caused the same decrease in the internal bonding strength (SBE), although the pitch content of the paper with stearic acid was significantly lower. In other words, the compound structure of stearic acid was more harmful for fiber–fiber bonding strength. Furthermore, the papers with cyclic pitch compounds β-sitosterol and abietic acid had a similar pitch content although the SBE decreased significantly more with abietic acid. This in turn means that the compound structure of abietic acid was more harmful for fiber–fiber bonding strength.

**Figure 7:**
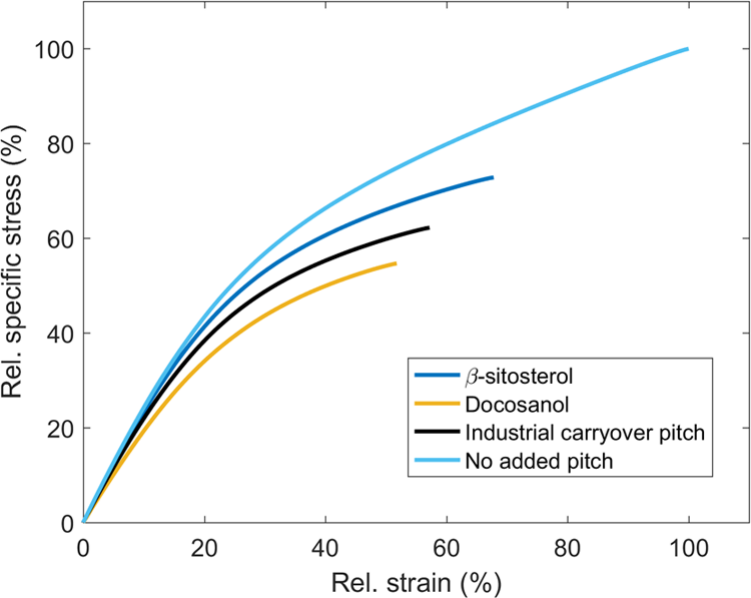
Influence of the addition of pitch to the papermaking suspension on the specific stress versus strain curve of Kraft paper. The curves are normalized against the rupture point of the paper without the addition of pitch, i.e., strain/breaking strain_pitch not added_ and specific stress/tensile index_pitch not added_.

The specific stress–strain curves shown in Figure 7 further illustrate the importance of fiber–fiber bonding strength on the tensile strength of paper. The addition of pitch compounds had practically no influence on the tensile stiffness (elastic behavior) of paper due to constant paper density. Slightly lower tensile stiffness occurred only in the case of the weakest paper with docosanol. After reaching the plastic region, i.e., the onset of fiber–fiber bond failure,^36^ differences in the specific stress–strain curves started to occur. Papers with weaker internal bonding strength reached the plastic region sooner and eventually ruptured at lower specific stress and strain values. The stress (TI) and strain at break of paper without the addition of pitch were 83.9 ± 1.6 Nm g^–1^ (95% confidence limits) and 2.66 ± 0.06%, respectively. These findings show similarities to those of Borodulina et al.^37^ and Seth and Page^38^ who concluded that the stress–strain curves of papers that differ only by the strength of fiber–fiber bonding (i.e., constant density) show identical behavior until close to rupture.

### 3.3 Influence of Individual Pitch Compounds on the Strength Increasing Effect of Cationic Starch

**Figure 8:**
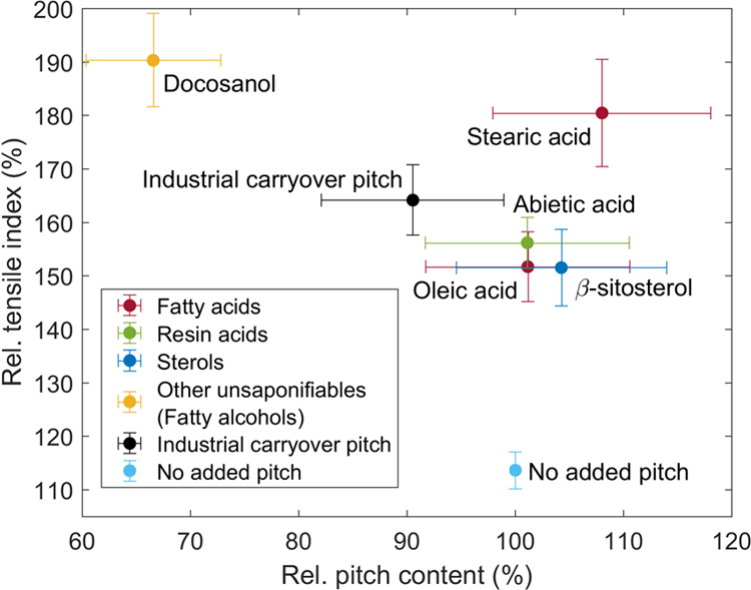
Influence of cationic (C) starch on pitch retention (pitch content, PC) and tensile index (TI) in Kraft paper. The values with cationic starch are normalized against the values without cationic starch but with the addition of pitch compounds, i.e., PC_C-starch added_/PC_C-starch not added_ and TI_C-starch added_/TI_C-starch not added_. Error bars are 95% confidence limits.

Very often, cationic starch is used as a strength agent in papers. Its proper functioning is potentially disturbed by complex formation with anionic CPEs (fatty/resin acids) and precipitation of CPEs on the fiber surface, which both impart fiber–fiber bonding (see Section 1). Thus, two questions are here of particular interest with respect to CPEs. First, if the effect of the individual CPE compounds on paper strength is the same in the presence of cationic starch. Second, if the cationic nature of starch increases the retention of negatively charged CPEs (fatty/resin acids) in the paper. Here, Figure 8 shows that the addition of cationic starch in most of the cases did not lead to a significant change in the retention of CPE compounds. Surprisingly, the retention of the fatty alcohol docosanol decreased.

Figure 8 also shows how the addition of cationic starch together with different pitch compounds influenced the TI of Kraft paper when compared to the influence of the respective pitch compounds without cationic starch. In other words, the strength increasing efficiency of cationic starch in the presence of different CPE compounds is described. Addition of cationic starch led to a moderate increase in the TI of handsheets with industrial carryover pitch in comparison to the increase with individual CPE compounds. The increase in TI was higher with the saturated linear compounds (stearic acid and docosanol) but not with the unsaturated linear compound (oleic acid). A lower increase in TI was in turn observed with the cyclic compounds (β-sitosterol and abietic acid) and oleic acid. Clearly, the lowest increase in TI was observed when the cationic starch was added without any additional CPEs.

**Figure 9:**
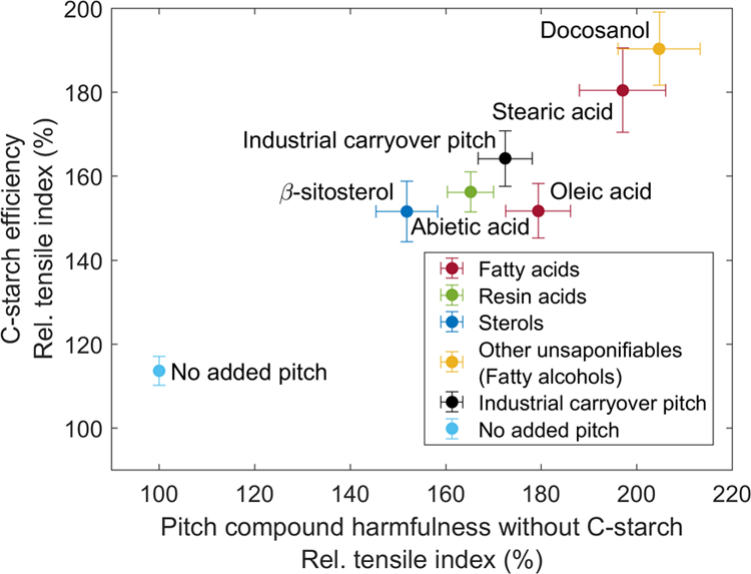
Influence of pitch compound harmfulness without cationic (C) starch (*x*-axis) on the efficiency of cationic starch in the presence of the respective pitch compound (*y*-axis). The cationic starch efficiency is taken from Figure 8. The values with cationic starch are normalized against the values without cationic starch but with the addition of pitch compounds, i.e., TI_C-starch added_/TI_C-starch not added_. The flipped ratio of the tensile indices (TI) shown in Figure 4 describes in turn the pitch compound harmfulness without C-starch, i.e., TI_pitch not added_/TI_pitch added_. Error bars are 95% confidence limits.

Figure 9 combines the TI results shown in Figures 4 and 8. The key aim is to investigate how the harmfulness of different CPE compounds without cationic starch influences the strength increasing efficiency of cationic starch in the presence of respective CPE compounds. Here, the pitch harmfulness without cationic starch is defined as the ratio of tensile strength without pitch to with pitch, i.e., the tensile index ratio shown in Figure 4 is flipped. The efficiency of cationic starch is in turn defined as the ratio of the tensile index with cationic starch to without cationic starch, in the presence of respective CPE compounds in both cases.

It was found that the higher the harmfulness of CPE compounds without cationic starch, the better the functioning of cationic starch as a dry strength agent in the presence of respective compounds. In other words, the efficiency of cationic starch was highest with docosanol and stearic acid because these components were the most harmful ones without cationic starch. Furthermore, the harmfulness of pitch compounds without cationic starch and the efficiency of cationic starch were linearly related. Only the influence of oleic acid varied from this trend, as it hindered the functioning of cationic starch more than its harmfulness without cationic starch suggested. Generally, these observations are in good agreement with those of Lindström et al.^39^ who stated that the strength increasing effect of starch is higher for weaker papers.

The addition of cationic starch together with different pitch compounds or without pitch did not influence the density of the prepared handsheets (see Figure S3 in the Supporting Information). Also, in the presence of cationic starch as a dry strength agent, no change in fiber–fiber bonding area (i.e., paper density) can be observed.

**Figure 10:**
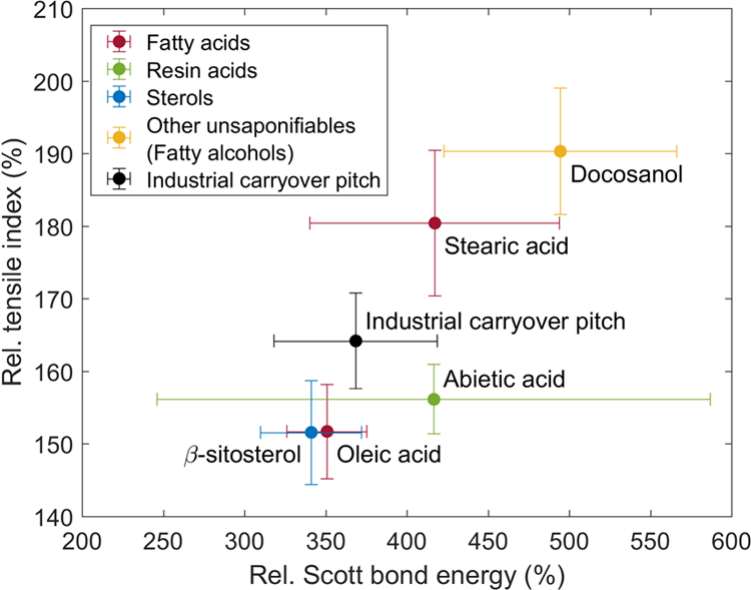
Influence of cationic (C) starch in the presence of pitch on Scott bond energy (SBE) and tensile index (TI) in Kraft paper. The values with cationic starch are normalized against the values without cationic starch but with the addition of pitch compounds, i.e., SBE_C-starch added_/SBE_C-starch not added_ and TI_C-starch added_/TI_C-starch not added_. Error bars are 95% confidence limits.

Figure 10 shows how the SBE increased significantly (by 350–500%) with TI by the addition of cationic starch together with different pitch compounds. Similar to TI, stearic acid and docosanol (saturated linear compounds) showed a higher increase in SBE in comparison to β-sitosterol and oleic acid. Both the SBE and TI with industrial carryover pitch were approximately in between these two groups. The analysis of SBE with abietic acid was in turn not precise enough due to the wide confidence interval (the number of replicates was only 3). Thus, in both cases, with and without starch as a dry strength agent, there are similar conclusions. Pitch is reducing the internal bond strength of the unbleached softwood Kraft paper without changing the bonded area of the fibers (sheet density), demonstrating that the strength of the adhesion in the bonded area of the fiber surfaces is disturbed by the presence of CPEs. As without C-starch, the rupture propagation of paper with C-starch was dominated by breaking of the fiber–fiber bonds and pulling out of the fibers from the paper structure (see Figure S2 in the Supporting Information).

**Figure 11:**
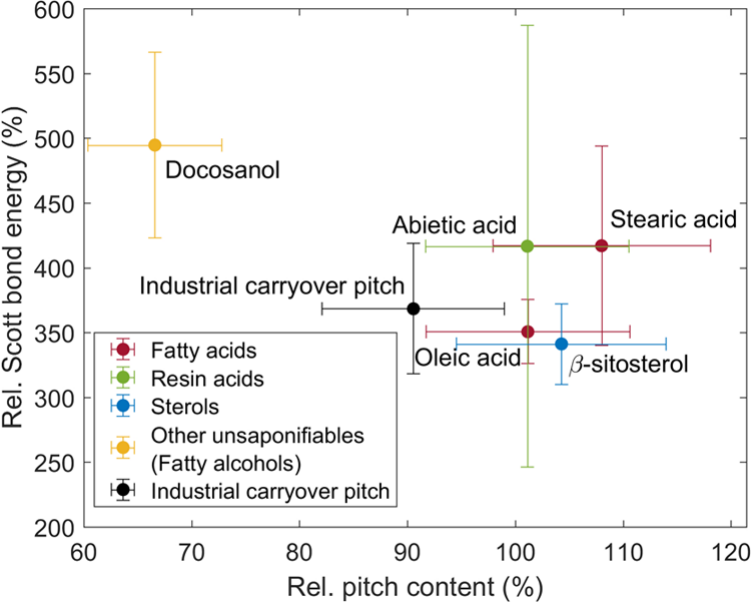
Influence of cationic (C) starch on pitch retention (pitch content, PC) and internal bond strength (Scott bond energy, SBE) in Kraft paper. The values with cationic starch are normalized against the values without cationic starch but with the addition of pitch compounds, i.e., PC_C-starch added_/PC_C-starch not added_ and SBE_C-starch added_/SBE_C-starch not added_. Error bars are 95% confidence limits.

The ability of cationic starch to improve fiber–fiber bonding strength in the presence of different CPE compounds varied considerably, although no significant or consistent changes occurred in the retention of pitch compounds, as shown in Figure 11. Docosanol was a clear exception, as its retention decreased together with the higher efficiency of cationic starch. These results mean that the structures of pitch compounds are important in defining the efficiency of cationic starch. In other words, the ability of cationic starch to improve fiber–fiber bonding strength is reduced most by β-sitosterol and oleic acid due to the structures of these compounds.

**Figure 12:**
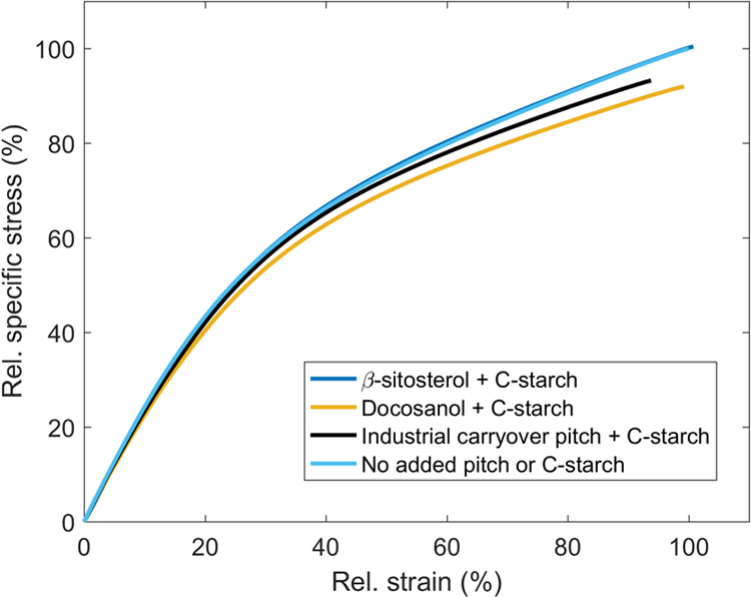
Influence of cationic (C) starch in the presence of pitch on specific stress versus strain curve of Kraft paper. The curves are normalized against the rupture point of the paper without the addition of pitch or C-starch, i.e., strain/breaking strain_pitch or C-starch not added_ and specific stress/tensile index_pitch or C-starch not added_.

As the specific stress–strain curves of papers without C-starch in Figure 7 illustrate, the curves in Figure 12 show that the influence of pitch compounds on the tensile stiffness (elastic behavior) was also negligible with C-starch due to constant paper density. The onset of fiber–fiber bond failure, i.e., beginning of the plastic region, occurred sooner for papers with weaker internal bonding strength, leading to rupture at lower specific stress. However, the differences in curves with C-starch were much smaller than without it, and the curves of paper without pitch/C-starch and paper with β-sitosterol/C-starch were identical.

**Figure 13:**
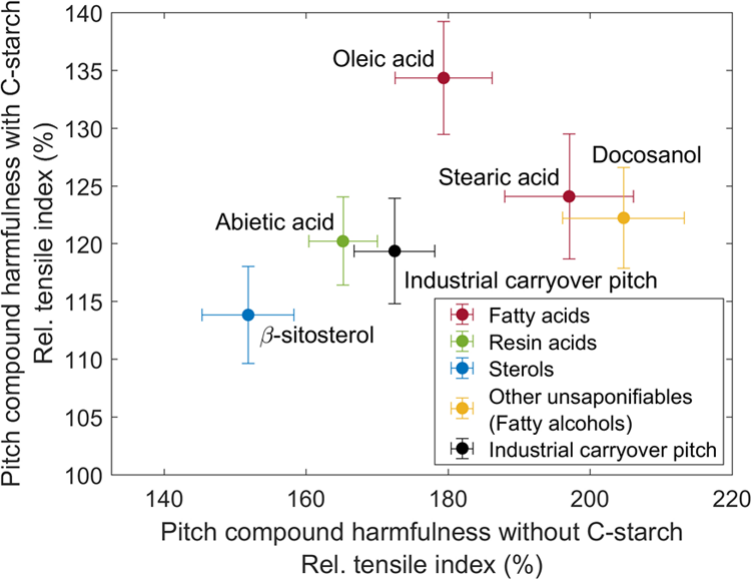
Harmfulness of pitch compounds on tensile strength without (*x*-axis) and with cationic (C) starch (*y*-axis). Harmfulness is defined as the ratio of tensile index TI without pitch to TI with pitch, i.e., TI_pitch not added_/TI_pitch added_ and TI_C-starch, pitch not added_/TI_C-starch, pitch added_. Error bars are 95% confidence limits.

Figure 13 shows the magnitude of the negative effect (the harmfulness) of pitch on paper strength, with and without C-starch. Again, harmfulness is defined as the ratio of tensile strength without and with pitch. It was found that the observed decrease in TI due to pitch was lower with cationic starch than without it. For example, the decrease was 16% with cationic starch and industrial carryover pitch, whereas a decrease of 42% was observed only with industrial carryover pitch. Even for oleic acid, which had performed worst with cationic starch, the relative strength loss is much higher without C-starch. Thus, it seems that cationic starch is an effective additive to mitigate strength losses due to CPEs. Besides precipitating on the fibers with the aid of alum, and thus blocking the anionic adsorption sites for cationic starch, the anionic CPEs (fatty/resin acids) also interfere by complex formation with cationic starch.

By further observing Figure 13, cationic starch evened out the differences between TI values of handsheets with different pitch compounds. This is also clearly seen by comparing the specific stress–strain curves shown in Figure 7 (without C-starch) and Figure 12 (with C-starch). In other words, the handsheets with stearic acid and docosanol (saturated linear compounds) as well as with abietic acid (cyclic compound) reached the same TI values as the ones with industrial carryover pitch. Although tensile strength was reduced with β-sitosterol (cyclic compound), the lowest negative influence of β-sitosterol on TI without cationic starch made it also the least harmful compound with cationic starch. Oleic acid (unsaturated linear compound) was in turn the most harmful compound, as it efficiently hindered the strength increasing effect of cationic starch, although its harmfulness without cationic starch was relatively high (see Figure 9). This combined with the facts that the portion of fatty acids from CPEs was the highest (see Figure 3) and they largely control the properties of pitch micelles makes oleic acid, and potentially all unsaturated fatty acids, most relevant CPE compounds contributing to the reduced performance of unbleached softwood Kraft paper.

## 4 Conclusions

In this work, we have investigated the detrimental effect of individual substances in carryover pitch extractives (CPEs) on the strength of paper from unbleached softwood Kraft pulp. Alum was used as a fixating agent, as it is used in industrial paper production, and the results were compared to industrial carryover pitch collected from the filtrate of the pulp entering the paper mill. Overall, a strength decrease of 34–52% was recorded for the different pitch compounds and industrial carryover pitch. The bonded fiber surface remained constant in the absence and presence of pitch, leading to the conclusion that CPEs impede the adhesion in the bonded fiber regions, thus causing a loss in bonding strength.

As a whole, the findings of the current work demonstrate that fatty acids, especially unsaturated ones (oleic acid), are the most relevant CPE compounds contributing to the reduced efficiency of cationic starch and decreased strength of unbleached softwood Kraft paper. This makes the cleaning of process waters by precipitating CPEs on the pulp fibers with the aid of retention agents such as alum harmful for paper strength.

The major result of this work is that fatty acids are the most relevant compounds present in CPEs. Fatty acids readily precipitate on fibers, thereby interfering in fiber–fiber bonding in handsheets; they form micelles that dissolve nonsaponifiable compounds, and in addition, they are the most abundant compounds.

Addition of cationic starch improved the paper strength and furthermore evened out the strength differences of papers with different pitch compounds. This means that the lower the strength without cationic starch, the more efficient the functioning of cationic starch. However, oleic acid (unsaturated fatty acid) was an exception, as it was above average harmful for paper strength without starch and also heavily impaired the functioning of cationic starch. This makes oleic acid, and potentially all unsaturated fatty acids, especially harmful for the strength of unbleached softwood Kraft paper.

## Supporting Information Available

Description of ISO standards; pitch/cationic starch influence on paper density; and microscopy image of the rupture line of paper (PDF)
