# Supplementary material for: How Different Carryover Pitch Extractive Components are Affecting Kraft Paper Strength
Source: ACS Omega. 2021 Oct 26;6(44):29350–9. doi: 10.1021/acsomega.1c02579 (PMC8581972; doi:10.1021/acsomega.1c02579)
Supplement: Supplementary file 1 — ao1c02579_si_001.pdf [file ao1c02579_si_001.pdf]

How different carryover pitch extractive components are affecting Kraft paper strength

Supporting information

Jussi Lahti[1,2], Roman Poschner[1,2], Werner Schlemmer[1], Andrea Hochegger[3], Erich Leitner[3], Stefan Spirk[1,2] and Ulrich Hirn[*,1,2]

[1]Institute of Bioproducts and Paper Technology, Graz University of Technology, Inffeldgasse 23, Graz 8010, Austria

[2]Christian Doppler Laboratory for Fiber Swelling and Paper Performance, Boltzmanngasse 20/1/3, Vienna 1090, Austria

[3]Institute of Analytical Chemistry and Food Chemistry, Graz University of Technology, Stremayrgasse 9/II, Graz 8010, Austria

[*]Corresponding Author:

Ulrich Hirn

Email: ulrich.hirn@tugraz.at

Description of ISO standards

- **ISO 14453** – determination of acetone-soluble matter: Soxhlet extraction of pulp/paper samples with acetone for 4 h. Evaporation of acetone after extraction followed by drying of the residual pitch under 105°C. Gravimetrical determination of pitch content in dry pulp/paper. Number of replicates 2.

- **ISO 5264-2** – PFI mill laboratory beating of pulp: Pulp with a consistency of 10% is beaten between a central rotor with bars and a surrounding refining housing with a smooth surface. Both the rotor and the housing are rotated to the same direction but with different peripheral speeds.

- **ISO 5267-1** – determination of pulp drainability with Schopper-Riegler method: A pulp suspension (1 L) of 0.2% concentration is filtered through a perforated screen plate into a funnel with bottom and side orifice. The °SR degree corresponds with the water volume discharged through the side orifice, i.e. a smaller water volume leads to a higher °SR degree. Number of replicates 2.

- **ISO 5269-2** – preparation of laboratory sheets for physical testing with a Rapid-Köthen method: A pulp suspension (783 mL) of 0.3% concentration is filtered through a mesh screen with the aid of suction to form a round paper sheet. The formed paper sheet is dryed (98°C & 15 min) under suction without being allowed to shrink. Number of replicates 4-5.

- **ISO 536** – determination of grammage: Measurement of paper area (A = 317 cm^2) and mass (m) followed by calculation of basis weight (m/A). Number of replicates 4-5.

- **ISO 534** – determination of thickness, density and specific volume: Paper thickness measured with a precision thickness measuring device. Number of replicates 11-15. Paper density calculated with the aid of thickness and basis weight (ISO 536).

- **ISO 1924-3** – determination of tensile properties: Tensile test of paper with rate of elongation of 100 mm/min. The test span is 100 mm and paper width 15 mm. Number of replicates 16-20.

- **ISO 16260** – determination of internal bond strength: Before measurement square paper sample (25.4 × 25.4 mm^2) is adhered to an anvil (lower side) and to an L-shaped platen (upper side) with a double-sided tape. The measurement is performed by allowing a pendulum to impact the L-shaped platen which leads to splitting (delamination) of the sample in thickness direction. After measurement the energy absorbed during delamination

(Scott bond energy) is calculated based on the over-swing of the pendulum after impact and the masses/dimensions of the system components. Number of replicates 9-15.

Supporting Figures

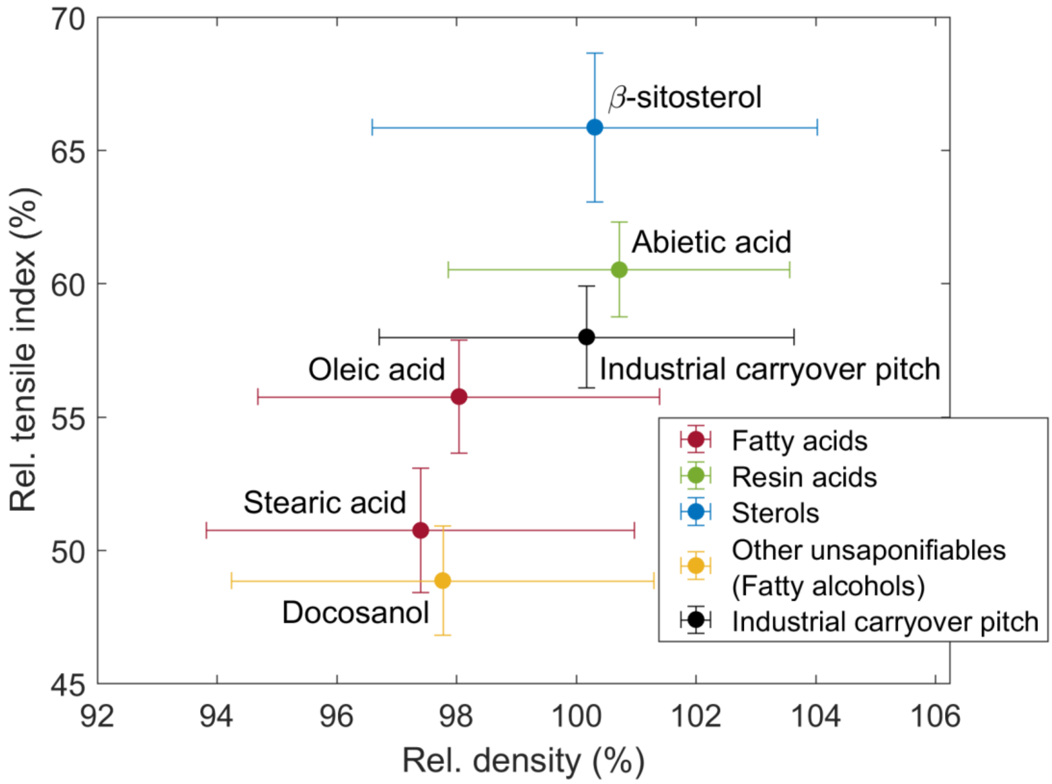

Figure S1: Small influence of pitch retention on Kraft paper density. Density (D) of handsheet vs. tensile index (TI), error bars are 95% confidence limits. The values with pitch addition are normalised against the value without pitch addition, i.e. $D_{\text{pitch added}}$ / $D_{\text{pitch not added}}$ and $TI_{\text{pitch added}}$ / $TI_{\text{pitch not added}}$.

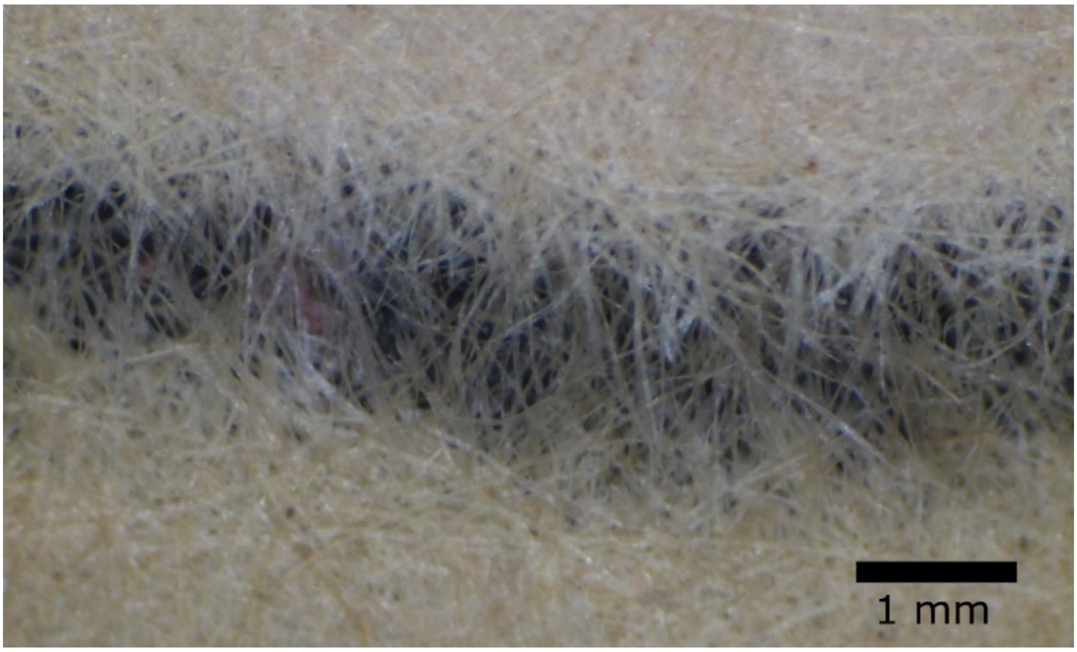

Figure S2: Optical microscope image of typical rupture line of paper after tensile testing. The rupture of all papers with/without added pitch compounds and C-starch propagated by breaking of the fiber-fiber bonds and pulling out of the fibers from the paper structure, i.e. the fibers themselves did not break.

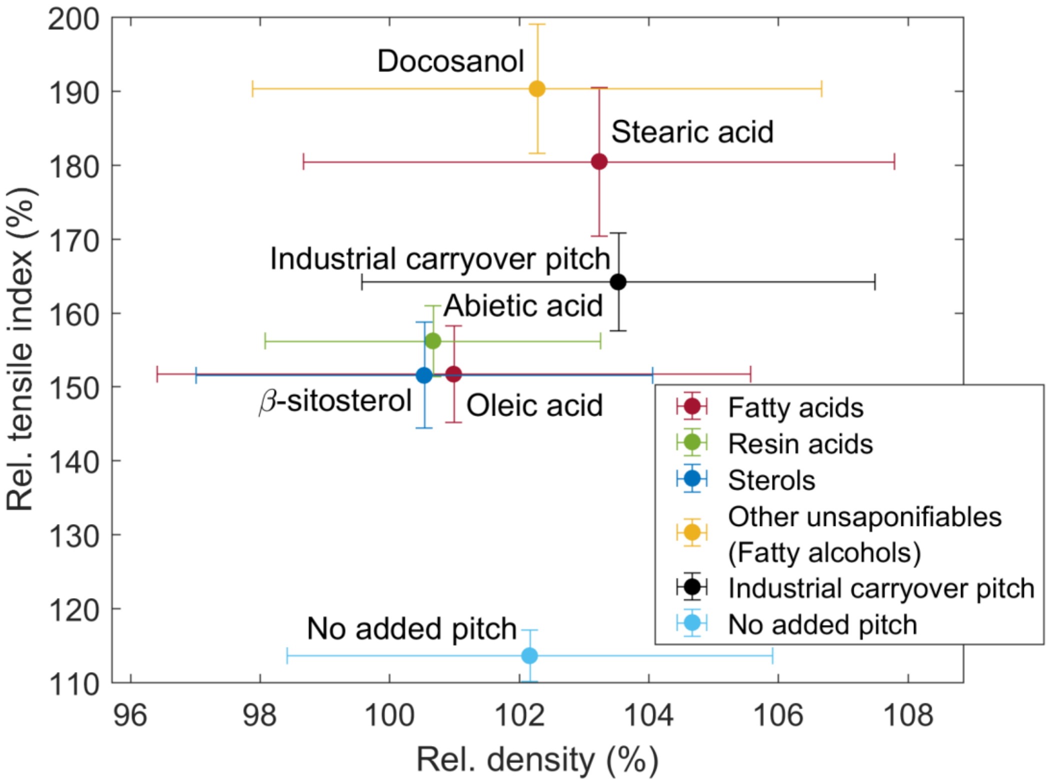

Figure S3: Influence of cationic (C) starch in the presence of pitch on density (D) and tensile index (TI) in Kraft paper. The values with cationic starch are normalised against the values without cationic starch but with the pitch compound added, i.e. $D_{\text{C-starch added}}$ / $D_{\text{C-starch not added}}$ and $TI_{\text{C-starch added}}$ / $TI_{\text{C-starch not added}}$. Error bars are 95% confidence limits.
